# Dual Targeting of Neuropilin-1 and Glucose Transporter for Efficient Fluorescence Imaging of Cancer

**DOI:** 10.1007/s11307-025-01993-7

**Published:** 2025-03-06

**Authors:** Jianwei Zhu, Can Zhou, Jian Yang, Zhenhua Wang

**Affiliations:** 1 Department of Gastroenterology, Jiading District Central Hospital Affiliated Shanghai University of Medicine & Health Sciences, Shanghai, 201800 China; 2 School of Medicine, Shanghai University, Shanghai, 200444 China

**Keywords:** Neuropilin-1 (NRP1), Glucose transporter 1 (GLUT1), Fluorescence imaging, Dual-targeting, Cancer

## Abstract

**Purpose:**

Early diagnosis and complete resection of cancer are pivotal for enhancing patient survival rates and prognosis. However, a significant current challenge lies in the lack of specific imaging probes for the identifying various tumor types. The expression levels of neuropilin-1 (NRP1) and glucose transporter 1 (GLUT1) in most tumors, including breast cancer, are closely linked to tumor proliferation and metastasis. This study seeks to develop a novel near-infrared fluorescence (NIRF) probe aimed at precise tumor detection by targeting NRP1 and GLUT1.

**Procedures:**

G_0_ was conjugated with N_3_-PEG_4_-ALKADK and 2-Azido-2-deoxy-D-glucose to synthesize the NGF probe. The spectral properties (fluorescence and absorbance spectra) of NGF were studied in both methanol and water. The targeting specificity of NGF towards NRP1 and GLUT1 was evaluated using confocal fluorescence microscopy imaging, flow cytometry assays and in vivo IVIS spectrum imaging.

**Results:**

A dual-targeting fluorescent probe named NGF was successfully synthesized to bind to both NRP1 and GLUT1 receptors. NGF exhibited greater hydrophilicity (Log *P* = -0.95 ± 0.07) and superior optical properties compared to its precursor, G_0_. Confocal fluorescence imaging, flow cytometry assays, and blocking studies revealed that the cellular uptake of NGF correlated with the NRP1 and GLUT1 expression levels across cell lines. Moreover, a strong linear relationship (R^2^ = 0.98) was observed between fluorescence intensity and increasing NGF concentrations in MDA-MB-231 cells. In vivo IVIS imaging in animal models demonstrated specific binding of NGF to breast cancer (MDA-MB-231) and colorectal cancer (HCT116), with prolonged retention observed up to 72 h.

**Conclusions:**

This study highlighted the efficient targeting and sustained retention of the dual-target heterodimeric fluorescent probe NGF, binding to NRP1 and GLUT1 receptors. These findings suggest significant potential for clinical applications in early cancer detection and fluorescence image-guided surgery.

**Supplementary Information:**

The online version contains supplementary material available at 10.1007/s11307-025-01993-7.

## Introduction

The incidence of malignant tumors is on the rise globally, with particular concern for breast cancer and gastrointestinal cancer [1–3]. Among females, breast cancer is the most prevalent, while gastrointestinal cancer ranks third in incidence [4, 5]. Complex and high-incidence cases like triple-negative breast cancer (TNBC), colorectal cancer (CRC), and gastric cancer pose significant challenges in diagnosis and treatment strategies [6, 7]. TNBC known for its aggressiveness, high recurrence rate, and poor prognosis, underscores the critical need for improved screening, diagnosis and treatment strategies [8, 9]. Therefore, early screening, diagnosis, and treatment strategies for breast cancer are crucially important, especially for TNBC patients [10, 11]. Similarly, the global impact of CRC cannot be underestimated. Patients with advanced CRC face a poor prognosis, with lower 5-year survival rates [12, 13]. Currently, colonoscopy remains preferred method for CRC screening, followed by the pathological biopsy for lesion confirmation. However, patient compliance remains challenging due to its adverse effects and semi-invasive nature [14]. More precise diagnostic techniques are urgently required to enhance screening rates and diagnostic accuracy, ultimately improving patient survival and quality of life.

Neuropilin-1 (NRP1) is a multifunctional type I transmembrane protein involved in a various physiological process, including cell signaling, proliferation, migration, and angiogenesis [15–17]. It plays a critical role in interacting with cytokines such as the vascular endothelial growth factor (VEGF) family and its receptor, as well as transforming growth factor-β1 (TGF-β1) and its receptor [18–20]. Disruption of interactions within the VEGF/NRP1 axis has the potential to impede the invasion and metastasis of cancer cells [21]. Moreover, NRP1 is upregulated in variety of tumor types, including TNBC and CRC. Developing targeted drugs against NRP1 is critical for inhibiting communication between cancer cells and their microenvironment, thereby restraining tumor growth and spread [22]. Therefore, accurate detection of NRP1 expression is particularly crucial for diagnosing malignant tumors like TNBC and CRC.

The glucose transporter 1 (GLUT1) plays a crucial role in facilitating glucose uptake in tumor cells to support their energy metabolism [23, 24]. Overexpression of GLUT1 is commonly observed in various cancers due to the high energy demands of rapidly dividing cancer cells [25–27]. This heightened expression of GLUT1 in tumor tissues enhances glucose uptake, providing a significant energy source for anaerobic glycolysis [28, 29]. Moreover, studies have demonstrated a synergistic interaction between the long non-coding RNA (lncRNA) GAL and GLUT1 in promoting the migration and invasion of colorectal cancer cells [25]. Overall, GLUT1 serves as an early indicator of carcinogenesis and presents potential opportunities for novel diagnostic and therapeutic approaches.

Fluorescence imaging technology has gained popularity in medical imaging due to its high sensitivity, rapid acquisition speed, and absence of ionizing radiation [30–33]. Recently, researchers have developed fluorescent molecular probes that target tumor receptors for both diagnostic imaging and surgical navigation [34–37]. For instance, Dang’s fluorescent probe targeting the α_v_β_3_ integrin receptor demonstrated precise tumor localization and guidance in breast cancer surgery [38]. Additionally, Dang developed an NIR fluorescent probe targeting ERβ/HDAC for diagnosing and treating prostate cancer [39]. Cyano dyes serve as an excellent platform for constructing tumor-targeted near-infrared probes due to their unique photophysical properties and potential for chemical modification [40].

**Fig. 1:**
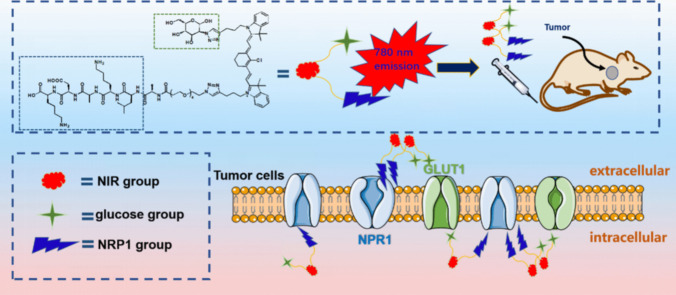
Proposed action mechanism of the near-infrared fluorescent probe **NGF**

Among the numerous tumor markers, NRP1 and GLUT1 have gained significant attention due to their close association with tumor occurrence and progression. This study aims to develop a fluorescent imaging molecular probe capable of effectively targeting both NRP1 and GLUT1. The dual-targeting fluorescent probe NGF was successfully synthesized through meticulous design and synthesis (Fig. 1). By incorporating 2-Azido-2-deoxy-D-glucose into the QS-1 [41], the probe’s water solubility was enhanced, improving its uptake at the tumor site. *In vitro* studies demonstrated the probe’s precise recognition and high binding affinity for NRP1 and GLUT1. In animal models, it enabled accurate localization and visualization of tumors through GLUT1- and NRP1-assisted ligand recognition. Hence, this probe stands as a valuable tool for the early cancer detection and treatment.

## Materials and Methods

### Synthesis of Probe NGF

Details for the synthesis and characterization of the probe NGF were provided in the Supporting Information.

### Optical Property

To investigate the spectral characteristics of probe, NGF (25 μM) was prepared using methanol, and the NGF stock solution was diluted to various concentrations (0.5, 1, 2, 3, and 4 μM) with both methanol or water as solvents. The absorption spectra at various concentrations were recorded using a UV–vis absorption spectrometer. To better understand how the solvent affects the fluorescence properties of NGF, the fluorescence emission spectra of 4 μM NGF were analyzed in both methanol and water. The molar absorption coefficient (*ε*), fluorescence quantum yield (*Φ*_*F*_), as well as the stability of NGF were investigated, and the details were provided in the Supporting Information.

### Cell Culture

MDA-MB-231 and NCI-H1299 cells were cultured in RPMI-1640 medium, while HCT116 cells were cultured in DMEM medium. All media were supplemented with 10% fetal bovine serum and 1% penicillin–streptomycin to promote optimal cellular growth. The cells were incubated in a humidified environment at a constant temperature of 37 °C with a CO_2_ concentration of 5%.

### Animal Models

The SPF female Balb/c nude mice (4–5 weeks old) were obtained from Changzhou Cavens Laboratory Animal Co., LTD (Changzhou, China). Tumor models were created by subcutaneously injecting 0.5 ~ 1 × 10^7^ MDA-MB-231, HCT116 or NCI-H1299 cells in 100 μL PBS into the right forelimb of the nude mice. Imaging studies were conducted once the tumor volume reached approximately 100 mm^3^. All animal experiments requiring the use of mice were approved by the animal care committee of Shanghai University (ECSHU 2024–004).

### Western Blot Analysis

Cancer cells MDA-MB-231, NCI-H1299 and HCT116 were lysed at 4 ℃ using RIPA lysis buffer containing 1% PMSF. Subsequently, the supernatant was collected by high-speed centrifugation, and proteins (50 μg) from different cells were separated by SDS-PAGE. Then, the resulting protein bands were transferred onto a polyvinylidene difluoride (PVDF) membrane and blocked nonspecific binding sites with 5% skim milk for 1 h at room temperature. The antibodies of NRP1 (Abcam, China, 1:1000) and GLUT1 (Beyotime, China, 1:1000) were incubated overnight at 4 ℃ with the PVDF membrane, respectively. A secondary antibody, rabbit IgG-HRP (1:1000) was added and incubated for 1 h at room temperature. Finally, the imprinted proteins were detected using the Chem-Doc XRS^+^ gel imaging system, the ImageJ software was used to perform quantitative analyses of NRP1 and GLUT1 protein expression in different cell lines.

### Confocal Fluorescence Microscopy Imaging

MDA-MB-231, HCT116, and NCI-H1299 cells in logarithmic growth phase were seeded in 6-well plate and incubated at 37 ℃ in a 5% CO_2_ humidified incubator for 12 h, then the cells were fixed with 4% paraformaldehyde containing 1% TritonX-100 overnight at 4 ℃. Next, cells were treated with NGF (2 μM) for 1 h, stained with DAPI for 5 min, and finally the fluorescent images were captured using laser scanning fluorescence confocal microscopy (Olympus IX51). Images were captured using a U-LH100 HG mercury lamp with a WIY (Cy5 channel) filter and a 200 ms exposure time. A 10 × eyepiece and a 20 × objective were used for a total magnification of 200 × . A scale bar of 50 μm was included for reference.

### Flow Cytometry

Cancer cells MDA-MB-231, NCI-H1299 and HCT116 in logarithmic growth phase were harvested and transferred to sterile tubes with DMEM medium at a density of 2 × 10^5^ cells per tube. Various concentrations of NGF (0.125, 0.25, 0.5, 1, and 2 μM) were added, and the cells were incubated in a humidified incubator with 5% CO_2_ at 37 ℃ for 1 h. Subsequently, the cells were resuspended in 0.5 mL of PBS buffer, and about 2 × 10^4^ target cells were extracted for flow cytometry analysis (FACS Verse, BD) with FL4 channel, and the mean fluorescence intensity was quantified using FlowJo software.

### Blocking Study

For the blocking study, the cells of HCT116, NCI-H1299, and MDA-MB-231 were pretreated with the D-glucose (50 μM), NRP1 peptide (50 μM), and D-glucose + NRP1 peptide (50 μM) for 30 min. Subsequently, the probe NGF (2 μM) was added and incubated for another 1 h. Then the fluorescence signals were detected by flow cytometry and microscopy imaging.

### *In vivo* Fluorescence Imaging

Animal fluorescence imaging was conducted using an IVIS Imaging spectroscopy system (PerkinElmer Inc., USA). MDA-MB-231, HCT116, and NCI-H1299 tumor-bearing mice (*n* = 3–5) were intravenously injected with 25 μM NGF (200 μL) via the tail vein. Following injection, mice were anesthetized with a 2% *v*/*v* isoflurane/oxygen mixture at a flow rate of 2 mL/min to maintain stable physiological conditions during imaging. Subsequently, long-term optical imaging observations of tumor-bearing mice were performed using the IVIS Spectrum imaging system (PerkinElmer, USA). Imaging scans were conducted at various time points (1, 2, 4, 6, 8, 12, 24, 36, 48, 60, and 72 h) after NGF administration. The excitation wavelength (*λ*_*ex*_) was set to 745 nm and the emission wavelength (*λ*_*em*_) was set to 840 nm for optimal fluorescence signal acquisition.

### Static Analysis

The data were presented as mean ± standard deviation (SD) for at least three independent assays. Statistical analyses were conducted using SPSS software with the t-test or one-way-ANOVA. A *P* value of less than 0.05 was considered statistically significant.

## Results

### Synthesis and Characterization of Probe NGF

Using a CuAAC reaction, we synthesized a novel probe named NGF by conjugating G_0_ with N_3_-PEG4-ALKADK and 2-Azido-2-deoxy-D-glucose. The synthetic route of NGF was showed in Figure S1. The characterization of NGF involved mass spectrometry, HPLC analyses, and ^1^H/^13^C NMR spectroscopy (Figures S2-S5). Mass spectrometry and ^1^H/^13^C NMR spectroscopy results confirmed that the molecular weight of NGF corresponded to its theoretical value, affirming the synthesis's accuracy. Furthermore, HPLC analysis confirmed the purity of NGF.

### Photophysical and Photochemical Properties

**Fig. 2:**
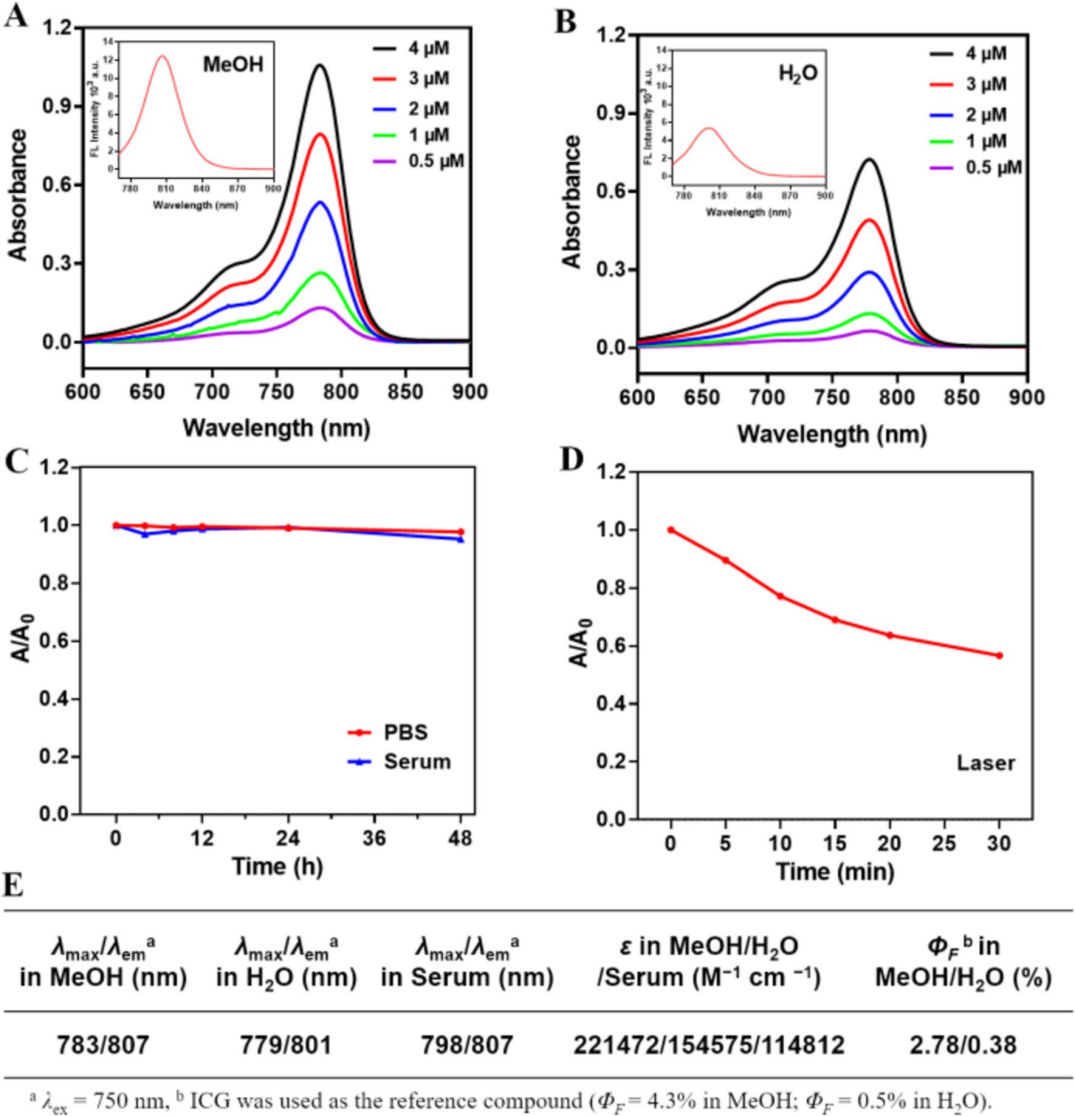
Optical properties of **NGF**. Absorbance spectra of probe **NGF **at different concentrations (0.5, 1, 2, 3 and 4 μM) in (**A**) MeOH and (**B**) H_2_O. (Inset) Fluorescence spectra of **NGF **at 4 μM in MeOH and H_2_O, respectively. The stability of **NGF **in PBS, 10% mouse serum (**C**) and laser (**D**). (**E**) Photophysical and photochemical properties of **NGF**

We investigated the optical properties of the NGF probe. Figure 2A illustrates that NGF exhibited a absorption peak at 783 nm, whereas G_0_ showed a peak at 780 nm. Regarding fluorescence emission, NGF peaked at 807 nm compared to 804 nm for G_0_. These slight wavelength differences indicate that incorporating targeting molecules N_3_-PEG_4_-ALKADK and 2-Azido-2-deoxy-D-glucose did not alter the core fluorescence characteristics of G_0_. Thus, NGF retains the original fluorescence properties of G_0_ while gaining new targeting capabilities, thereby enhancing its specificity for specific biological applications. Further analysis revealed that a 4 μM solution of NGF in methanol exhibited a maximum absorbance of 1.06 and a fluorescence intensity of 12365 a.u., comparable to those observed for the G_0_ fluorophore.

The absorbance and fluorescence intensity ratios measured in water (H_2_O) were notably lower compared to those in methanol (MeOH), indicating some degree of aggregation of NGF in aqueous solutions. As compared with QS-1 [41], the maximum absorbance of both 4 μM NGF and QS-1 solutions showed consistency between MeOH and H_2_O conditions (Figs. 2A-B). However, NGF exhibited a slightly higher maximum fluorescence intensity of 5374 a.u. in H_2_O compared to QS-1. This finding suggests that the inclusion of 2-Azido-2-deoxy-D-glucose improved the solubility of NGF in water, enhancing its hydrophilicity, as evidenced by a Log *P* value of −0.95 ± 0.07.

Additionally, the photo, serum and aqueous stability of NFG were tested. As can be seen from Figs. 2C-D, NGF demonstrated strong stability in both PBS and 10% mouse serum. After 48 h of incubation in these solutions, the absorbance of NGF was recorded at 97.7% and 95.2% of the initial absorbance (*A*_*0*_), respectively. Furthermore, after being irradiated with a laser for 30 min, the absorbance of NGF decreased to 56.7% of *A*_*0*_. All the results indicate that NGF possesses strong stability and is suitable for subsequent cell and animal experiments.

Meanwhile, the molar absorption coefficient (*ε*) and fluorescence quantum yield (*Φ*_*F*_) of NGF were investigated. The results (Fig. 2E) showed a significant difference between in MeOH and H_2_O, which might be attributed to the partial aggregation of NGF in aqueous solution. The photophysical properties of NGF under serum conditions were also investigated, and the results showed that the *ε* of NGF was significantly lower than that observed in MeOH, this phenomenon might be due to the conjugation of NGF with albumin in serum. In summary, NGF functions as an innovative cyanogen probe with enhanced hydrophilicity and improved optical properties.

### *In vitro* Binding Specificity

**Fig. 3:**
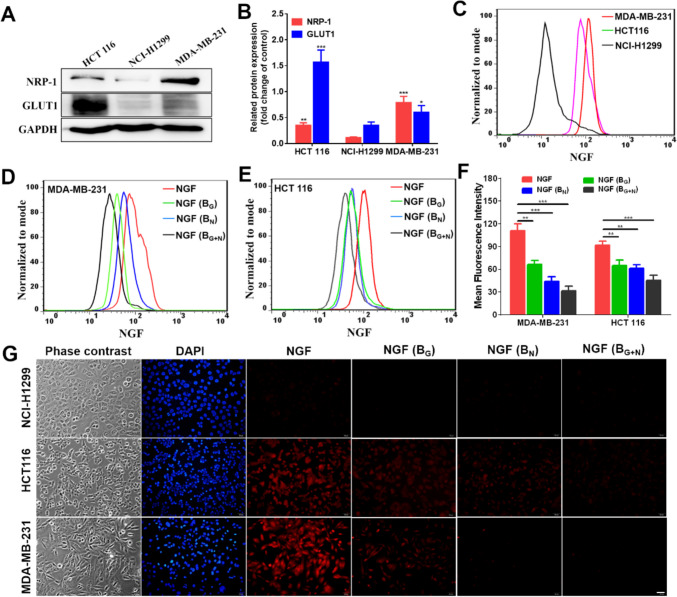
Binding specificity of **NGF** *in vitro*. (**A**) Western blot analysis of NRP1 and GLUT1 expression in different cancer cell lines HCT116, NCI-H1299 and MDA-MB-231. (**B**) Quantification analysis of western blot data by ImageJ software, *P < 0.05, **P < 0.01, ***P < 0.001 (*vs.* NCI-H1299 cells). (**C**) Flow cytometry analysis of **NGF** (2 μM) incubated with different cancer cell lines MDA-MB-231, HCT116 and NCI-H1299. (**D**) Flow cytometry analysis of **NGF** (2 μM) in cancer cells MDA-MB-231 with different treatment. (**E**) Flow cytometry analysis of **NGF** (2 μM) in cancer cells HCT116 with different treatment. (**F**) The quantification analysis of mean fluorescence intensity by FlowJo software. (**G**) Confocal fluorescence microscopy imaging of MDA-MB-231, HCT116 and NCI-H1299 cells incubated with **NGF** (2 μM) or blocking (scale bar = 50 μm). B_G_, B_N_, and B_G+N_ indicated the cells were blocked by D-glucose (50 μM), NRP1 peptide (50 μM), and D-glucose + NRP1 peptide (50 μM), respectively. *P < 0.05, **P < 0.01, ***P < 0.001

Prior to investigating the targeting specificity of the probe, we evaluated the expression of NRP1 and GLUT1 in various cancer cells. Figures 3A-B indicate that MDA-MB-231 cells exhibited higher levels of NRP1 protein compared to the other cell lines studied, and high level of GLUT1 expression was observed in HCT116 cells, whereas NRP1 and GLUT1 were minimally expressed in NCI-H1299 cancer cells, this suggests that the cancer cell lines MDA-MB-231, HCT116 and NCI-H1299 can be utilized to assess the binding specificity of NGF for NRP1 and GLUT1.

We investigated the specific targeting capability of the NGF probe towards NRP1 and GLUT1 using flow cytometry and microscopy imaging experiments in MDA-MB-231, HCT116, and NCI-H1299 cells. The flow cytometry illustrated in Fig. 3C showed that NGF was accumulated significantly in HCT116 and MDA-MB-231 cells than that in NCI-H1299 cells, indicating the probe NGF can specifically target cancer cells with high NRP1 and GLUT1 expression. Meanwhile, the Fig. 3G shows that upon incubation with MDA-MB-231 cells, NGF was significantly internalized, displaying intense fluorescence at non-nuclear locations within the cellular structure. This result highlights NGF's strong targeting affinity for NRP1-positive MDA-MB-231 cells. Similarly, substantial internalization was observed in HCT116 cells, albeit with a weaker fluorescence signal compared to MDA-MB-231 cells, indicating NGF's specificity towards GLUT1, and its correlation with GLUT1 expression levels. In contrast, minimal fluorescence was detected in NRP1- and GLUT1-negative NCI-H1299 cells, underscoring NGF's selective targeting of NRP1-positive tumor cells. Flow cytometry and fluorescence imaging experiments confirmed that NGF effectively targets tumor cells expressing high levels of NRP1 and GLUT1, aligning with their respective expression profiles.

In addition, a blocking study of NGF was conducted by pre-treating MDA-MB-231 and HCT116 cells with the D-glucose (50 μM), NRP1 peptide (50 μM), and D-glucose + NRP1 peptide (50 μM) for 30 min. The results illustrated in Figs. 3E-G showed that the fluorescence signals can be blocked by the D-glucose, NRP1 peptide, and D-glucose + NRP1 peptide. According to the quantitative analysis of flow cytometry, the uptake of dual cancer receptor targeting probe in MDA-MB-231 and HCT116 was 1.7–2.5 and 1.4–1.5 times higher, respectively, compared to mono-receptor block conditions. These results indicating that probe NGF exhibited high specificity for both NRP1 and GLUT1 *in vitro*.

**Fig. 4:**
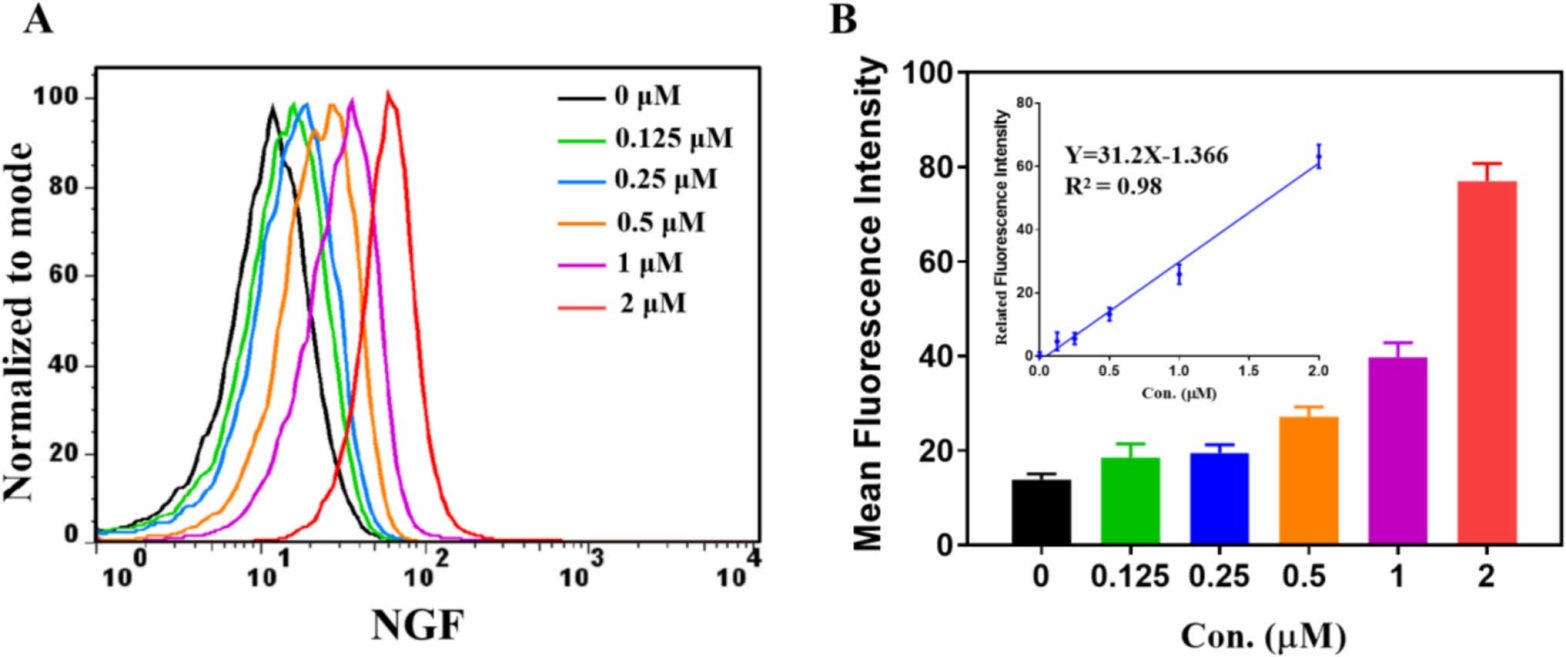
The cellular uptake of **NGF **in MDA-MB-231 cancer cells. (**A**) Flow cytometry analysis of different concentrations of **NGF **incubated with MDA-MB-231 cancer cells for 1 h. (**B**) The quantification analysis of mean fluorescence intensity by FlowJo software

Furthermore, we used flow cytometry to explore the relationship between cellular uptake in MDA-MB-231 cells and the concentration of NGF probe. Figure 4 demonstrates a clear and direct correlation between the fluorescence intensity of MDA-MB-231 cells and increasing concentrations of NGF, exhibiting a strong linear relationship (R^2^ = 0.98). These findings provide robust confirmation of NGF's specific binding capability to tumor cells expressing high levels of NRP1, while also establishing a quantitative association between fluorescence intensity and NGF concentration.

### Fluorescence Imaging of Tumor-Bearing Mice

**Fig. 5:**
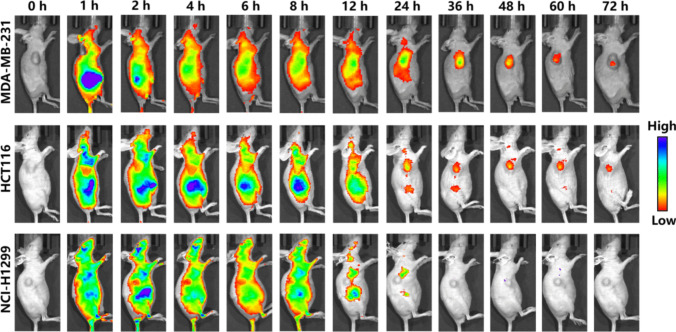
NIR fluorescence images of **NGF** (25 μM, 200 μL) in MDA-MB-231, HCT116 and NCI-H1299 tumor-bearing mice (*n* = 3–5)

To evaluate the probe's targeting efficacy towards NRP1 *in vivo*, we selected MDA-MB-231, HCT116, and NCI-H1299 tumor-bearing mouse models, each representing different levels of NRP1 and GLUT1 expression. As shown in Fig. 5, the tumor of MDA-MB-231 and HCT116 mice could be clearly identified at 1 h post-injection, indicating the probe's tumor-targeting capability. In contrast, fluorescence intensity was comparatively weak in NCI-H1299 tumors. Importantly, while the fluorescent signal cleared from non-target organs within 12 h, it persisted at the tumor site for up to 72 h. The long half-life of NGF in blood and tumor was found to be associated with serum binding by SDS-PAGE analysis (Figure S7). This sustained and specific fluorescence signal underscores the NGF probe's potential for tumor diagnosis. Significantly, after 24 h, only the tumor site exhibited a fluorescent signal, further confirming the probe's excellent tumor-specific imaging capability.

## Discussion

Due to tumor heterogeneity and complex tumor-stroma interactions, dual-target molecular probes exhibit higher selection specificity and detection sensitivity compared to mono-target molecular probes [42]. Numerous studies have demonstrated the advantages of employing the dual-target strategy to construct bispecific molecular probes, which include multivalent interactions, optimized molecular sizes, improved affinity, enhanced tumor targeting specificity, and excellent pharmacokinetics [43]. Chen et al*.* designed and developed ^68^ Ga- and ^18^F- labeled molecular tracers targeting the FAP and α_v_β_3_ dual-specific heterodimer FAPI-RGD, demonstrating its superiority over mono targets in human pancreatic cancer tumor-bearing mouse model. The performance of this tracer not only enhances tumor uptake and targeting specificity but also extends tumor retention time. Thus, this radioactive tracer holds significant clinical potential for patients with tumors expressing FAP and integrins [44, 45]. It is evident that research on dual-target radioactive tracers represents a promising direction for future development.

Dual-target NIRF imaging represents a novel approach for visualizing gastrointestinal tumors and breast cancer *in vivo*, providing valuable insights into cancer detection and precision diagnosis. The overexpression of NRP1 and GLUT1 in various tumors makes them promising targets for accurate cancer diagnosis. Peptides are considered excellent candidates for developing dual-labeled imaging agents due to their inherent specificity. Studies have shown that bispecific conjugates exhibit superior tumor uptake capabilities compare to single-targeted agents, suggesting potential applications of heterodimeric structures in imaging and therapeutic contexts [46].

Intraoperative fluorescence visualization technology is crucial for the advancing of surgical navigation. This imaging technology allows the real-time identification and localization of tumors, blood vessels, and nerves during surgery, providing a valuable tool for precise targeting and optimal surgical planning. By applying fluorescent labels on the tumor tissue, surgeons can clearly visualize its location and boundaries during procedures. This not only lowers the risk of surgical complications, but also improves the survival rates and quality of life.

This study introduces a novel dual-target fluorescent imaging probe, NGF, designed to target NRP1 and GLUT1. G_0_ serves as the fluorophore and is conjugated with a tumor-targeting peptide for NRP1. In addition, 2-Azido-2-deoxy-D-glucose was incorporated as a co-ligand to enhance water solubility and improve the targeting efficacy. And NGF exhibited excellent photophysical properties and stability. NGF’s excellent performance makes it a promising fluorescent molecular probe for tumor imaging and intraoperative navigation.

The UV absorption peak and fluorescence emission peak serve as crucial benchmarks for evaluating the performance of fluorescent probes. Minor deviations observed in these peaks between the dual-targeted fluorescent probe NGF and the fluorophore G_0_ indicated that the incorporation of targeting molecules ALKADK and 2-Azido-2-deoxy-D-glucose did not alter the fundamental fluorescence properties of G_0_. Furthermore, noteworthy findings showed that both the absorbance and fluorescence intensity of NGF were significantly higher in methanol compared to water at equivalent concentrations. And the fluorescence intensity of NGF in H_2_O was higher than that of QS-1 [41]. Meanwhile, NGF possesses strong photo, serum and aqueous stability, which is suitable for subsequent *in vitro* and *in vivo* investigations. These results establish NGF as an innovative cyanine probe with enhanced hydrophilicity and superior optical properties.

To assess the *in vitro* targeted binding ability of NGF, MDA-MB-231, HCT116, and NCI-H1299 cell lines with different expression of NRP1 and GLUT1 were utilized. Western blot analysis and confocal fluorescence imaging revealed that NGF exhibited enhanced targeting efficacy towards MDA-MB-231 and HCT116 cell lines, which have higher expression levels of NRP1 and GLUT1 compared to the NCI-H1299 cell line with lower expression levels of these receptors. Significant fluorescence signals were specifically detected in MDA-MB-231 and HCT116 cells, indicating NGF’s specific internalization into cells overexpressing NRP1 and GLUT1 via receptor targeting. Additionally, flow cytometry analysis revealed NGF uptake by MDA-MB-231 cells was concentration-dependent.

NIRF imaging further confirmed the targeting ability and prolonged retention of NGF in MDA-MB-231 and HCT116 tumors, characterized by high NRP1 and GLUT1 expression, lasting at least 72 h post-injection, which attributed to the specifically conjugation of the meso-Cl in NGF to cysteine [47–49]. There findings underscore NGF’s potential in developing fluorescent probes for intraoperative tumor navigation. Notably, NIRF imaging detected fluorescence signals at the tumor site within one hour injection, providing clear visualization of malignant tumors that persisted for up to 72 h. Therefore, NGF shows potential as a dual-targeting fluorescent probe suitable for advanced applications in biomedical imaging.

## Conclusions

This study illustrated the effective targeting and prolonged retention of the dual-labeled heterodimeric fluorescent molecular probe NGF, which targets NRP1 and GLUT1 in breast and colorectal cancer tumor-bearing mice. The ability of NGF to precisely locate and retain in tumor tissue offers substantial groundwork for future diagnostic and therapeutic applications. These findings underscore the benefits of dual-targeted receptor probes in accurately identifying tumors and introduce promising avenues for intraoperative surgical guidance.

## Supplementary Information

Below is the link to the electronic supplementary material.

Supplementary file1 (DOCX 2460 KB)

## Data Availability

All data produced or examined in this research are contained within this published article and its supplementary materials. If additional data files are required in an alternative format, they can be obtained from the corresponding author upon a reasonable request.
