# Supplementary material for: Dual Targeting of Neuropilin-1 and Glucose Transporter for Efficient Fluorescence Imaging of Cancer
Source: Mol Imaging Biol. 2025 Mar 6;27(2):250–9. doi: 10.1007/s11307-025-01993-7 (PMC12062102; doi:10.1007/s11307-025-01993-7)
Supplement: Supplementary file 1 — Supplementary file1 (DOCX 2460 KB) [file 11307_2025_1993_MOESM1_ESM.docx]

***Supporting Information***

**Dual targeting of neuropilin-1 and glucose transporter for efficient** **fluorescence imaging of Cancer**

Jianwei Zhu^1^, Can Zhou^2^, Jian Yang^2*^, Zhenhua Wang^1*^

1. Department of Gastroenterology, Jiading District Central Hospital Affiliated Shanghai University of Medicine & Health Sciences, Shanghai, 201899, China;
2. School of Medicine, Shanghai University, Shanghai, 200444, China.

^*^Corresponding author: Jian Yang, Email: yj_scu@163.com; Zhenhua Wang, Email: zhenhuaw@sjtu.edu.cn

**Contents**

[**1. Supplementary Methods 3**](#_Toc183174674)

[1.1 Materials 3](#_Toc183174675)

[1.2 Synthesis of the intermediate QS-1 3](#_Toc183174676)

[1.3 Synthesis of probe NGF 4](#_Toc183174677)

[1.4 The lipid-water partition coefficient (Log *P*) of NGF 4](#_Toc183174678)

[1.5 The photophysical properties of the probe NGF 5](#_Toc183174679)

[1.6 Serum and aqueous stability of probe NGF 5](#_Toc183174680)

[**2. Supplementary Figures 6**](#_Toc183174681)

[Figure S1. 6](#_Toc183174682)

[Figure S2 6](#_Toc183174683)

[Figure S3. 7](#_Toc183174684)

[Figure S4 7](#_Toc183174685)

[Figure S5. 8](#_Toc183174686)

[Figure S6. 8](#_Toc183174687)

[Figure S7.. 9](#_Toc183174688)

1. Supplementary Methods

1.1 Materials

All reagents and solvents were purchased from Aladdin or Energy Chemical unless otherwise described. 2,3,3-Trimethylindolenine and 5-Iodopent-1-yne was obtained from Haohong Biomedical Technology Company Limited (Shanghai, China). 15-Azido-4,7,10,13-tetraoxapentadecanoic acid was obtained from Aikon Biomedical Technology Company Limited (Suzhou, China). The human breast cancer cell line MDA-MB-231, the human colon cancer cell line HCT116, and the human non-small cell lung cancer cell line NCI-H1299 were obtained from Cell Bank of Chinese Academy of Sciences (Shanghai, China).

Mass spectrometer (MS) was recorded on a Waters SQ-detector 2 mass spectrometer (USA). HPLC analysis was performed with a UV detector (Waters 2487, USA) and C18 column (250 mm × 4.6 mm, 10 μm, Phenomenex, China). The fluorescence spectra were operated on a Photon technology international QM/TM fluorescence spectrometer (USA). The absorption spectra were collected on a Perkin Elmer Lambda 25 UV/Vis spectrophotometer (USA). Fluorescence imaging of cells was carried out by Olympus IX51 confocal microscope (Tokyo, Japan). Fluorescence imaging of mice was done on an IVIS Lumina XR small animal optical in vivo imaging system (USA).

1.2 Synthesis of the intermediate QS-1

The synthetic route of QS-1 was according to the reported literature [1]. In brief, CuSO_4_·5H_2_O (7.88 mg, 0.032 mmol, 0.5 eq), L-ascorbate sodium (12.48 mg, 0.063 mmol, 1 eq), and THPTA (13.69 mg, 0.032 mmol, 0.5 eq) were dissolved in H_2_O followed by the addition of G_0_ (45 mg, 0.063 mmol, 1 eq). Subsequently, N_3_-PEG_4_-ALKADK (34.67 mg, 0.038 mmol, 0.6 eq) dissolved in a mixed solution of DMF and H_2_O was added dropwise to the reaction mixture, and stirred for 1.5 h under N_2_ protection at 45℃. The product was separated and purified using semi-preparative HPLC followed by drying with vacuum freeze dryer, resulting in the acquisition of a dark green solid QS-1 (25 mg).

1.3 Synthesis of probe NGF

The CuSO_4_·5H_2_O (1.53 mg, 0.006 mmol), L-ascorbic acid sodium salt (2.43 mg, 0.012 mmol), and THPTA ligand (2.66 mg, 0.006 mmol) were dissolved in H_2_O, followed by the addition of QS-1 (20 mg, 0.012 mmol) and 2-Azido-2-deoxy-D-glucose (2.51 mg, 0.012 mmol). Then, DMF was added to the reaction system at a ratio of 5 : 1 with H_2_O at 45 ℃ under inert gas protection for 1.5 h. After completion of the reaction, the crude product was separated and purified using semi-preparative HPLC, and subsequently dried using a vacuum freeze dryer to yield **NGF** as dark green solid (10 mg) in a yield of 47.6%. The HPLC analysis revealed a product purity exceeding 98%. ^1^H NMR (500 MHz, D_2_O) δ 8.06 – 7.39 (m, 4H), 7.03 (d, *J* = 83.5 Hz, 8H), 5.95 (br, 1H), 5.17 (s, 1H), 4.97 (d, *J* = 7.5 Hz, 1H), 4.57 – 4.46 (m, 2H), 4.30 (s, 2H), 4.12 (ddd, *J* = 28.8, 17.7, 7.9 Hz, 7H), 3.98 (s, 2H), 3.85 – 3.72 (m, 2H), 3.65 (br, 4H), 3.47 (m, *J* = 20.4 Hz, 4H), 3.38 – 3.20 (m, 11H), 2.82 (t, *J* = 7.4 Hz, 4H), 2.79 – 2.43 (m, 7H), 2.44 – 1.97 (m, 7H), 1.89 (s, 3H), 1.77 – 1.46 (m, 10H), 1.44 – 0.97 (m, 24H), 0.62 (dd, *J* = 24.7, 5.0 Hz, 6H). ^13^C NMR (126 MHz, D_2_O) δ 175.13, 175.01, 174.48, 174.41, 173.60, 173.51, 173.29, 171.87, 162.85, 162.57, 123.49, 119.78, 117.47, 115.14, 94.47, 76.14, 73.64, 69.50, 69.42, 69.38, 68.76, 68.69, 68.66, 66.51, 60.67, 53.41, 52.58, 52.17, 49.91, 49.73, 49.57, 49.51, 39.42, 39.03, 35.40, 35.31, 30.25, 30.39, 30.20, 29.84, 27.63, 27.59, 27.55, 27.51, 27.47, 27.46, 27.41, 27.36, 26.58, 26.17, 26.11, 26.06, 25.99, 25.37, 24.30, 24.20, 22.08, 21.89, 21.84, 21.77, 20.70, 20.66, 16.41, 16.36.

1.4 The lipid-water partition coefficient (Log *P*) of NGF

Probe **NGF** (10 μM) was added to a mixture of H_2_O (0.5 mL) and n-octanol (0.5 mL), shaken thoroughly, and centrifuged for 3 min. The absorbance (*A*) of NGF in both H_2_O and n-octanol was measured using a UV-vis absorption spectrometer. The concentrations of **NGF** in the two phases were calculated according to Lambert-Beer's Law *A* = *εCL*, and the Log *P* of **NGF** was determined using the equation Log *P* = log (*C_o_*/*C_w_*), in which the extinction coefficients (*ε*) of **NGF** in n-octanol and water are 124367 M^-1^ cm^-1^ and 154575 M^-1^ cm^-1^, respectively, the optical path length L is 1 cm, *C_o_* represents the concentration of the probe in octanol, and *C_w_* represents the concentration of the probe in water.

1.5 The photophysical properties of the probe NGF

Based on the absorption spectra of **NGF** at different concentrations, the molar absorption coefficient (*ε*) was calculated by fitting a straight line to the relationship between absorbance (*A*) and concentration (*C*). The fluorescence quantum yield (*Φ_F_*) of **NGF** in methanol and water was calculated according to the following equation [2]:

$$\Phi_{x}=\Phi_{\mathrm{st}}\times\frac{I_{x}}{I_{st}}\times\left( \frac{\eta_{x}}{\eta_{st}} \right)^{2}\times\frac{A_{st}}{A_{x}}$$

where, *I_x_* is the measured integrated emission intensity; *η* is the refractive index of the solvent; *A* is the absorbance of the solution; the subscript *x* denotes the probe; *st* denotes the reference compound.

1.6 Serum and aqueous stability of probe NGF

**NGF** solution (3 μM) was prepared in PBS and 10% (*v*/*v*) mouse serum, and the initial absorbance (*A_0_*) was measured. Then **NGF** was incubated with PBS and mouse serum at 37°C, and the absorbance (*A*) was recorded at 4, 8, 12, 24, and 48 h, respectively. The stability of the probe was evaluated based on the ratio of *A*/*A_0_*. For the photostability, **NGF** solution (3 μM) was prepared in methanol, and the initial absorbance (*A_0_*) was measured. Then **NGF** was then subjected to laser irradiation, and the absorbance measurements (*A*) were recorded at 5, 10, 15, 20, and 30 min, respectively. The stability of the probe was evaluated based on the ratio of *A*/*A_0_*.

**Reference**

[1] Qin S, Liu Q, Li K et al (2023) Neuropilin 1-targeted near-infrared fluorescence probes for tumor diagnosis. Bioorg Med Chem Lett 84:129196.

[2] Sun B, Zhao B, Wang D et al (2016) Fluorescent non-conjugated polymer dots for targeted cell imaging. Nanoscale 8:9837-9841.

2. Supplementary Figures

Figure S1. Synthetic route of NGF.


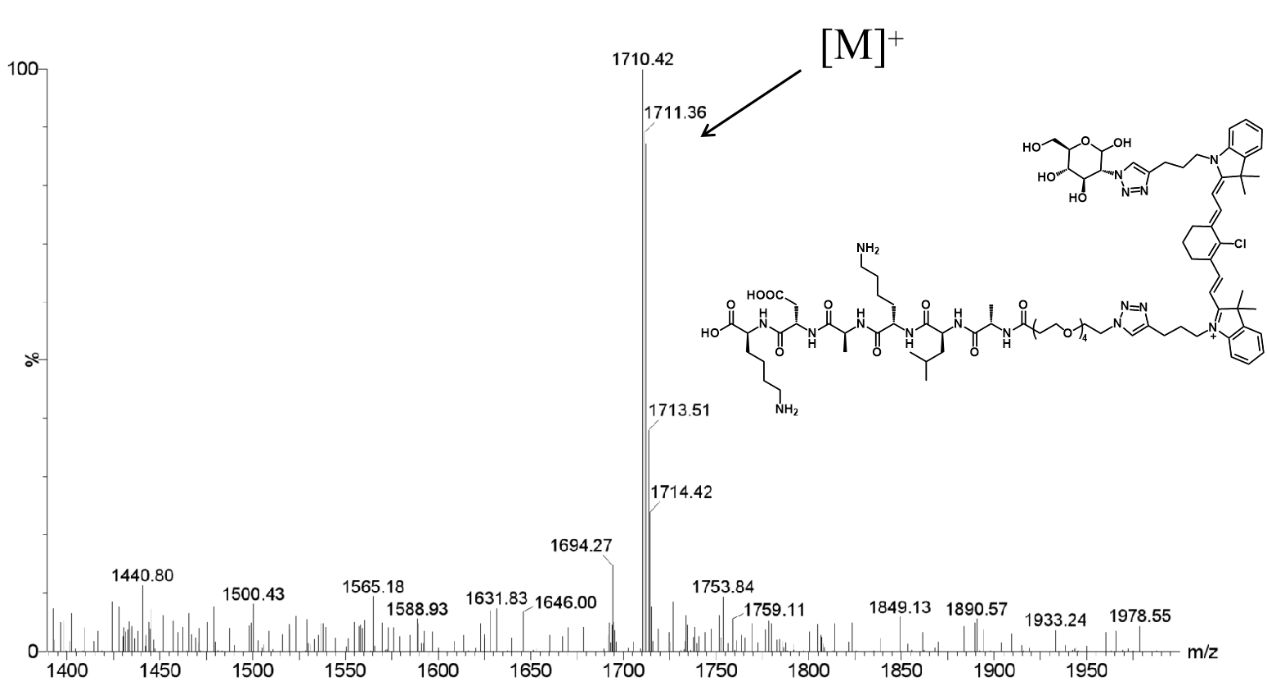


Figure S2. LC-MS (ESI Scan) of NGF.


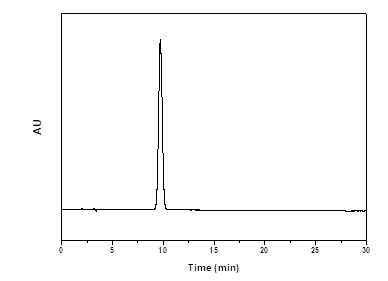


Figure S3. HPLC analysis of compound NGF at 254 nm.


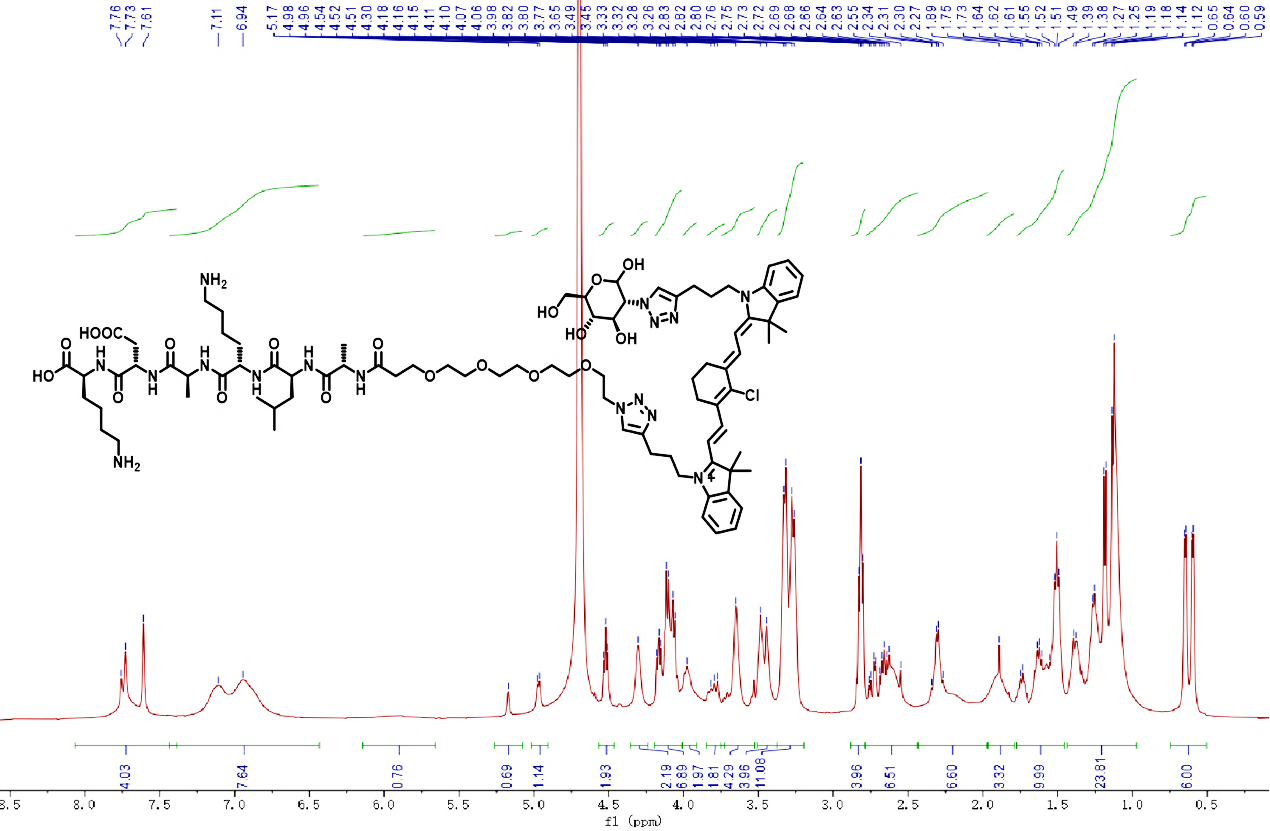


Figure S4. ^1^H-NMR spectrum of compound NGF (D_2_O).


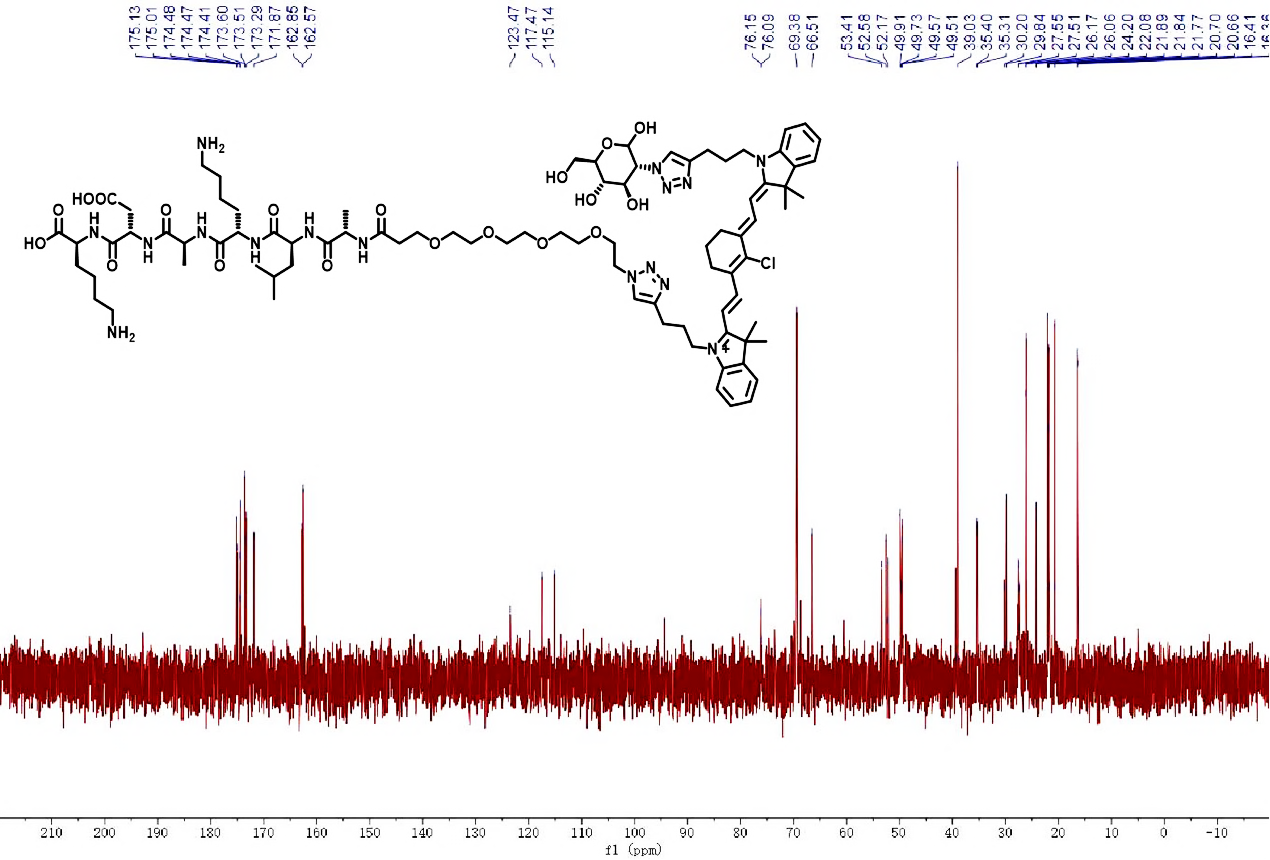


Figure S5. ^13^C-NMR spectrum of NGF (D_2_O).


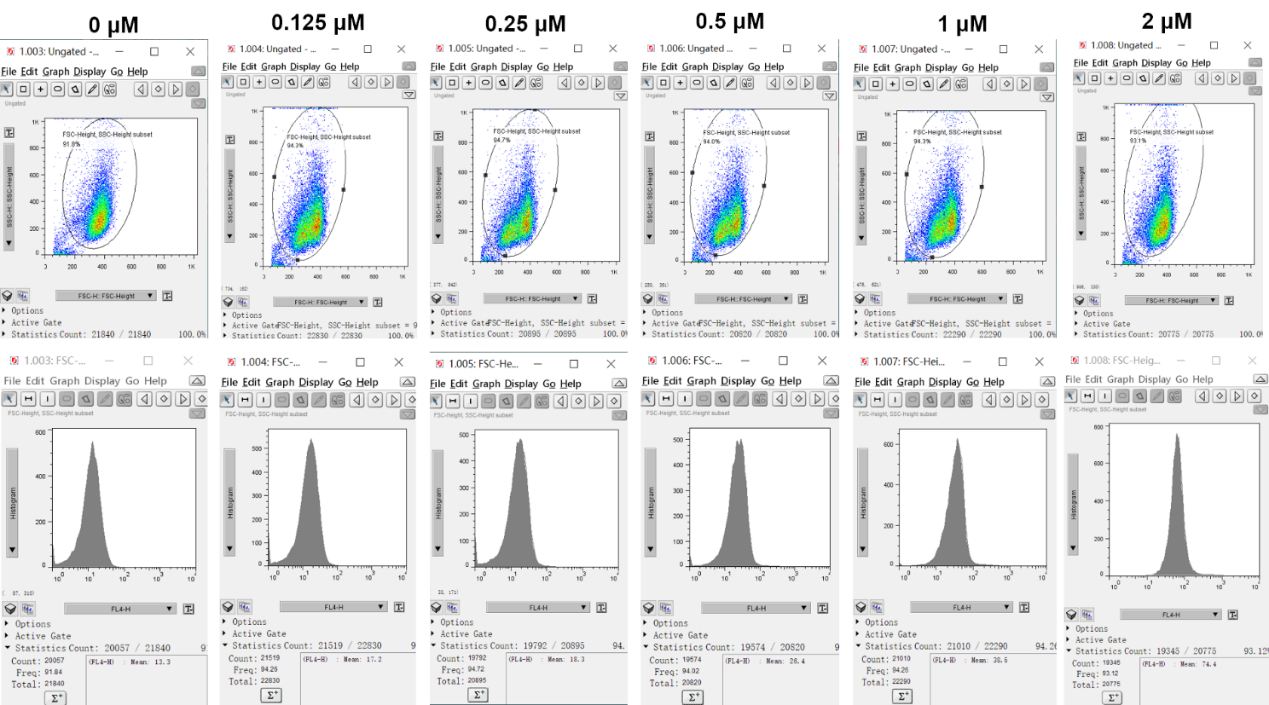


Figure S6. The information on gating and representative images for Figure 4A.


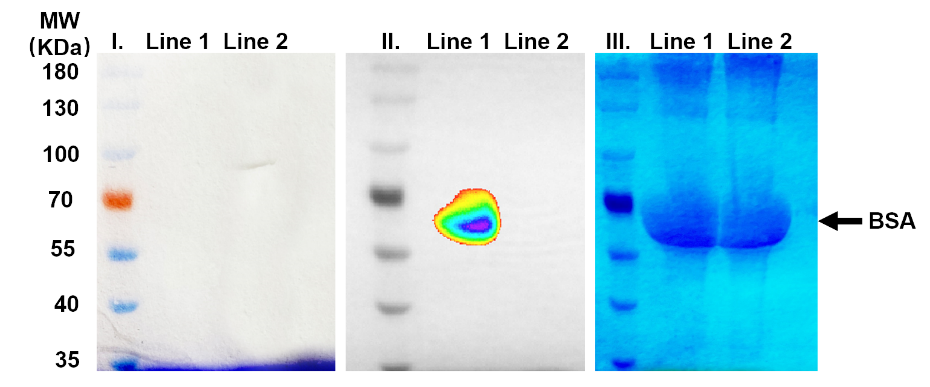


Figure S7. SDS-PAGE analysis of the reaction mixture (line 1) and free BSA (line 2) conducted under white light (I), fluorescence photography (II) with IVIS Imaging spectroscopy system, and after Coomassie blue staining (III).
